# Practical Points in Filling

**Published:** 1906-07-15

**Authors:** H. P. Martin

**Affiliations:** Windsor, Canada


### PRACTICAL POINTS IN FILLING.

BY H. P. MARTIN, D.D.S., WINDSOR, CANADA.

Read before the Detroit Dental Society, March 8th, 1902.

At the request of the Executive Committee I have culled the followiug comments on filling teeth from the current journals and present them for your consideration.

Occlusal Step.—I am a great advocate of the occlusal step in proximal cavities. From the standpoint of convenience I find it an excellent thing, especially in the distal cavities of the molars, by extending a right angle step we can see into the cavity and be sure of a strong wall, as it is difficult to get the anchorage in the buccal and lingual walls sufficiently strong and safe without some danger of tipping. —D. M. Gallie, Chicago.

Cement Retention.—Several years of careful experiment and observation have convinced me that the right quantity of cement mixed to a proper consistency placed beneath gold foil in a cavity and subjected to a pressure sufficient to weld the gold, will so perfectly seal the dental tubuli that we may entirely prevent the black line of discoloration so prevalent between cohesive gold and the tooth substance when the cavity is unlined. I prepare all cavities the same as if no cement were used, not relying in the least measure on the cement anchorage to hold the finished filling in place. I mix the cement to such a consistency that a small particle of it will quite readily drop from a common wire broach, and with this broach I carry it into the cavity. The reason for using the broach is because the amount that clings to it is so small that I am enabled to carry it to the floor of the cavity without smearing the walls. In this way a thin film of cement may be spread over the entire dentinal surface and the cavity is then ready for the filling.—F. S. Trickey, Freeport, Ills.

Failure of Fillings.—There are many things that enter into the question of "Failure of Fillings." Imperfect preparation of cavities is by far the largest factor in the problem. The dentist who practices extension for prevention, either in the extreme form or with modifications to suit the cases, can trace the recurrence of decay to faulty preparation. Possibly in order to avoid a display of gold or to save time, to save pain to the patient or from laziness and a desire to do the easy thing rather than the best, a compromise was made which compromise only postpones the day of reckoning, and this is especially true in the preparation of cavities.—W. A. Hoover, Gibson City, Ills.

Proximal Cavities.—When, in excavating a compound cavity in a tooth, you find a spot upon the next tooth oopposite the cavity, if the patient is young and the caries active, that should be cut out at once and the cavity filled. On the other hand, if the patient is comparatively old and one in whose mouth the progress of caries is stopping, I believe you are not jusitfied in cutting it out. You should take into consideration the different conditions you find in each individual mouth.—F. B. Noyes, Chicago.—*Review.*

A Source of Failure in Approximal Fillings.—The removal of unsupported enamel is an absolute necessity, whether upon the occlusal surface, upon the buccal or lingual walls, or upon the gingival margin, not only because it has not sufficient strength to withstand the stress of mastication, but because it does not possess sufficient strength to build and condense gold against without fracturing or breaking under the force of condensation.—C. F. Wallace, Erie, Pa.

Preparing Sensitive Cavities.—The first application of the burr can be made absolutely painless in the most highly sensitive cavities, simply taking "ethyl chloride" on the burr point and bringing it quickly into contact with the tooth.—J. M. Gale.

A Water-tight Gold Filling.—Prepare cavity as for an inlay and then give a retention form either by grooves or dovetails. Dry the cavity and line with thin cement.

Cut a piece of No. 30 or No. 40 gold foil as though for taking an impression for an inlay and put it down in the cavity in the usual impression method. This gives a cavity lined with gold, with cement in every possible irregularity.

Fill with any cohesive gold which the operator is in the habit of using, beginning first with the undercuts where the lining may be split, condensing thoroughly with hand plugger and finish in the usual way.

The method is applicable to the smallest gold filling or the largest contour, and makes gold fillings easy and sure.

Polish Amalgam Fillings.—The surface of an amalgam filling should be as carefully polished as any other filling. It can be done with fine pumice and Moose hide disks, followed by whiting and a fine brush wheel. This will produce a surface like burnished silver.

Silver Nitrate in the treatment of Children's Teeth.—In case the child presents as a patient with no caries existing, but deep fissures and roughened surface. are found inviting decay, silver nitrate should be used liberally. Have the solution made with distilled water. Heat a few shreds of asbestos wool to a white heat to drive off all organic matter and wind into a platinum wire or an iridio-platinum broach. Isolate the tooth with the rubber dam and then paint the tooth crown thoroughly with the solution With a quill pick or iridio-platinum broach work the liquid to the bottom of the fissures. Let it dry in the tooth if possible, but if unable to do so, take up all excess with bibulous paper or absorbent cotton. If allowed to dry in the crown, remove the salts with bibulous paper or absorbent cotton before removing the rubber dam.

This treatment twice each year with the ordinary daily cleaning by the patient is more than likely to absolutely inhibit caries in the mouth. The silver nitrate will not stain the enamel. It will stain organic matter in the enamel, but that may readily be removed with a little powdered pumice.

The silver nitrate destroys all bacterial life and renders exposed dentine immune to caries for months.—G. E. Hunt, of Indianapolis.

Condense Gold from the Center Towards the Margins.—In condensing gold we should always condense from the center to the cavity wall. If we begin the condensation next to the cavity wall and work toward the center, the tendency will be to draw the gold from the cavity wall, where it is already hardened under the force of the mallet blows, and all the malleting you can do will not place it in contact with the cavity wall (as it would have been had the condensation begun in the center), and a better margin will be made by finishing the malleting at the cavity wall. Care should be exercised not to use the pluggers on the enamel margin until there is sufficient gold beneath the plugger point, so that when the blow is made, the enamel will not be shattered.—J. L. Williams.

Failure of Fillings in the Proximal Surfaces of Bicuspids and Molars.—Among the various causes of failures due to the preparation of the cavities in proximal surfaces of molars and bicuspids is the failure to have a flat gingival seat upon which the filling may rest. The reason for this should be very apparent on account of the great stress brought upon it during mastication. The cavity should also be extended bucco-lingually in order to have the fillings exposed on both, the buccal and lingual surfaces. We cannot do better than to follow out to the letter Dr. Black's method of preparing cavities and finishing fillings which he so ably and thoroughly demonstrated to us at our last meeting. I trust these quotations may serve to stimulate our thought and provoke a good discussion on this everyday and important subject.

### DISCUSSION.

Dr. N. S. Hoff: There is one suggestion made in the report of Dr. Martin, in regard to the direction in which we should mallet foil in the cavity of the tooth that I would criticise. I do not believe it is the right thing to do to mallet foil towards the center, or away from the walls of the cavity. I condense towards the walls, and pay very little attention to the center of the filling. I think it is a mistake to get the center hard and full before you get the walls and margins protected. In the way suggested there is a possibility of bridging over spaces and not getting absolute contact of the gold with the cavity walls. Carry the filling along as nearly level as you can. If it is an incisal cavity, of course carry the lingual wall further along than the labial wall. In using cohesive gold there is not much spreading of it; it is not like the non-cohesive foil which you can force out against the walls of the cavity. The margins, grooves and surface contours should be most thoroughly condensed, but the center of the filling has no need for either hardness or density.

Dr. Martin: When you put in a pellet of gold the idea I drew from this paper was that you should put the pellet in the center of the cavity and commence from the center and work outwards, and by this means you might have it as level as you please without raising the center of it.

In illustrating this point it was suggested that you take a hot bar of iron and hammer it on an anvil; if you start in the center the ends will turn up, and if you hammer from the ends toward the center the iron will remain flat.

Dr. Spalding: The subjects of prophylaxis and pyorrhea is very near to my heart, and as Dr. Bowles spoke of it, it seems as if the term of pyorrhea alveolaris is used too indiscriminately. It has taken me a long time to know when to use it. It seems as if it is used for nearly all inflammations of the mouth, but it appears to me that if we used it only in connection with pus we shall have the whole significance of the term. There are so many varieties of cases that I do not try to classify the causes, but certainly there are systemic tendencies which make pyorrhea or inflammations of the mouth in one person more susceptible than another. It seems to me that imperfect occlusion certainly invites inflammation in the peridental membrane. Teeth do not cleanse themselves normally. Teeth having long crowns are much more liable to inflammations than short ones, because there is more surface to collect the putrefyable materials. I do not know of a case of normal occlusion where I have seen pyorrhea but nearly always where there is pyorrhea about the teeth, we find that there is abnormal occlusion, or the teeth get stress from a direction which nature did not intend them to have. Mal-occlusion developes a suitable place for the accumulation of fermentable materials. If you will investigate yourself, in cleansing your teeth, you will find that you neglect the lower incisors generally, and if you will watch yourself closely you will note that you brush your upper teeth vigorously but the lower incisors are woefully neglected. It is a difficult thing to teach patients to cleanse the incisors perfectly.

DR. N. S. HOFF: A patient came to me with every tooth in the mouth affected with a calcular deposits around the gum margins; but only two of these, so far as I could determine, were so badly affected that there was any pus in the pockets. The teeth all had the same physical appearance and looked the same as far as the gums were concerned. There was about the same amount of tartar on all the teeth. Was that a case of pyorrhea? I consider it so, although it is a limited case, and with two or three treatments I am satisfied I can cure it.

DR. SPALDING: Such cases are generally accepted as pyorrhea. It seems as if we have got to make some other term to fit those conditions. because we really must have a flowing pus. We ought to investigate it closely and, if possible invent some other term for this condition. You find pus very infrequently in the mouth, anyway, and the amount is usually small. It seems as if the term itself has been invented to described conditions where there is a flowing of pus, and we ought to have some other term to distinguish successive stages. From the first hyperemic condition of the gums to the total loss of the teeth it seems as if it is a case of degree. I do not think anyone could object to calling the case Dr. Hoff cites one of pyorrhea.

Dr. Bowles: I have accepted for some time the definition of pyorrhea for cases where there was pus flowing. I do not think we have a right to call an accumulation of pus around the teeth a suppuration, if there is no pus made there, because we are calling it what it is not. In reference to the infectious nature of pyorrhea, if I am not very greatly mistaken, Dr. Talbot, said in the discussion that followed the paper from which I quoted, that he had made numerous experiments trying to infect certain teeth with the pus obtained from others, and that he failed to do so, and could not produce pyorrhea where it was not already present.

There is a question that came up in my reading that I should like to have discussed in reference to the alkaline mouth washes. We used to hear it said that where there was an acid condition the thing to stop it was to administer an acid. Is that logical?

Dr. M. T. Watson: I think the homeopathic treatment of an abnormal acid condition would be the administration of an acid drug, but the local treatment of the mouth with mouth washes is hardly looked upon as curative; it is merely to overcome the injurious effects of your acid secretions rather than to correct the cause of your trouble. It has occurred to me in thinking of this subject of oral hygiene, and particularly in relation to the work that is being taken up in the public schools, that we should naturally expect the private schools, convents and places where children of the wealthy are looked after would take up this work first; yet, as a matter of fact, some of the most neglected cases I have ever seen have been children from well-to-do homes who are pupils in the very best private schools. It strikes me as particularly unfortunate that these children, whose parents are doing everything in their power to preserve their children's health, should consent to their remaining in school from 8:30 a. m., to late in the afternoon without an opportunity to brush their teeth. It seems to me that it might be well for us to take up this work in some of our private schools and convents, as well as with that class of people who are not quite so appreciative of hygenic methods. Some of the saddest cases of neglect that I have ever seen were children who had been in a convent where the physical care of the children is looked after with the greatest care, so far as the sisters know, and yet the need of proper dental attention was apparently never properly impressed upon them. Within a few days I have seen a case of a girl fifteen or sixteen years of age, who has lost some five or six teeth while in a convent. The teeth became troublesome, and, unknown to her parents, the child was hurried to a dentist and the teeth extracted. The result of such work as this is very sad, to say the least. I should be very glad, indeed, to see something done that would bring this matter to the attention of the authorities in private schools and convents, as well as in our public schools.

DR. J. S. HALL: Dr. Bowles asked concerning the application of alkaline washes in an acid condition. I read an article a few months ago in which it was stated that the more alkaline mouth washes that were used, the greater would be the development of acid conditions in the mouth. Like the development of muscle, the more exercise the more it developes. Alkaline mouth washes change acid saliva to alkaline, but they cannot influence the generation of acid saliva of the glands. It has occurred to me that the soft deposits around the necks of the teeth that seems so difficult for the patient to remove with the tooth brush, and which have to be scraped off, when allowed to stay in the mouth, are pushed down under the free margin of the gum at meal time and then they are difficult to remove. If it remains it decays, as the dentist may know by its odor when it is removed, and it causes an inflammation and it is difficult to tell in this decayed condition, whether it is pus or soft deposit. I have had a number of cases like this come to my attention where it was very difficult to diagnose. In my practice, I preach the gospel of the quil tooth-pick, tell them to keep them and get them between the teeth and use them; children the same. The quill tooth-pick makes a great difference in the condition of the teeth, on account of its sharp edge it will scale off the soft deposit where the wooden one will not.

In the article that Dr. Bowles quoted and its discussion, I think it was before the Central Society of New Jersey, they did not seem to know what pyorrhea was. Some of them attributed it to syphilis, others, if I remember correctly, attributed it to kidney disease; one man went so far as to say that invariably where Bright's disease was present, he always found pyorrhea also.

In connection with the scheme for this program for reading and preserving valuable suggestions found in the Dental Periodical, I have an index and under the different letters of it, I put the names of all articles I wish to preserve or refer to. For instance, under "P" put pyorrhea, filling material under "F." For periodical references put down opposite "I" for "*Items of Interest*," and 5'05 for May, 1905, and Reg. for *Register*, etc. In that way I keep in good arrangement all good articles I read and can turn to any article at a moment's notice, by looking at my index. I keep this in a regular book index, such as is used for ledgers.

Dr. Thompson: Dr. Hall has stirred me up a little by his remarks on the toothpick. If there is anything that stirrs my Irish, it is to have a patient come in with a toothpick sticking in between his teeth and say: There, Dock, there is the place, in there." One man was always doing that and I told him to fire that quil toothpick and never use it again. I gave him a brush, some silk floss and he went away saying that he would not use a quil toothpick again.

Dr. Talbot describes a condition in one of his articles in the *American System of Dentistry*, where you might pass your finger along the gum line and a slight exudate would be visible. A great many of us might rub our fingers along the gums of certain individuals and get that and call it pyorrhea, when it would not be pyorrhea. I had my mouth examined by one of our dentists and he told me I had pyorrhea around three teeth. Then I went to our friend Spalding and Dr. Rogers and they said I had all the causes of pyorrhea but did not have it.

Dr. D. M. Graham: Regarding the alkaline mouth wash it might provoke more acid theoretically; practically it does not. Theoretically we can digest proteids with pepsin and hydrochloric acid; practically pepsin is not much used for indigestion. I think it makes little difference whether your tooth wash is slightly alkaline or slightly acid. There is no question but what we get very good and most decidedly good results from proper application of a tooth wash, but I maintain that good results are got, not from its chemical but from its mechanical action. We should teach patients to properly wash the mouth with whatever preparations we prescribe. How long does your patient keep his mouthwash in contact with the tissues or tooth structure, a very short fraction of the time necessary. A great part of tooth structure is covered with a scale of something and possibly the actual tooth structure is not reached at all, and the saliva is, of course, normally alk aline. Nearly all the fluids of the body except the gastric juices are alkaline, and while we have an acid condition of the mouth it generally comes from lactic acid formation. It is acid because the saliva is acid. This might bring up the question as to the proper use of uric acid. Uric acid has been accused of some dire diseases. Examine the text books for the past five years and you will find that one of the causes always given for rheumatism is uric acid. Our present text books exclude it. It is not given as one of the causes for rheumatism now. Now lactic acid is given as a reason. While there is lactic acid present in the blood the question is, is that the cause? It is more probable that it is a result, and not a cause. It is a result of the breaking down of the tissues. We have several examples, and I think the saddest part of it is that we are obliged to read so much in the journals that is absolute rot; we must discriminate what is right and what is rotten. It seems that those who know least about the actual working of things write most to our confusion.

It is not more than fifteen years ago that a claim was made that peroxide of hydrogen, which had an occasional free oxygen, could be used with decided benefit by taking in internally, but it was found out that they were wrong, for the blood has several times more oxygen than it can use, so that it is not necessary to introduce any more oxygen into the system in order that the cells of the body may profit thereby. What in theory might appeal to us as being quite the correct thing will not apply in practice, as it is a different thing.

I have seen it mentioned in the dental journals that magnesium salts was an ideal antiseptic. A few months ago several papers were written and claims were made that it could be used as a local anesthetic and as a general anesthetic magnesium salts were the best thing we had. I have tried them, but am not prepared to say whether it is much good or not, because as with many other drugs, we know that we can get a practical anesthetic effect. It can be sterilized without deterioration, it is almost absolutely harmless and it is worth giving a fair trial. Adrenalin chloride is dangerous, as quite a few deaths have occurred lately from its use with cocain. The magnesium sulphate is now used mostly as an intra-spinal. I saw one case of leg amputation with the use of intra-spinal injection of the magnesium sulphate and with good results, but it is necessary to strengthen the analgesic action by a little chloroform. It is practically harmless and is worth our while to try.

Dr. Spalding: In reference to using alkaline washes in the mouth, I do not think they accomplish a great deal, neither do I think that an antiseptic that we use does much towards sterilizing the mouth; we must use mechanical means. In reference to the alkalinity, I think if Dr. Graham will try some case of a very sensitive cervical margin with bicarbonate of soda in the tooth powder, the patient will not complain of those sensitive places longer. The saliva in cases of sensitive teeth is not usually normal. By using something that will make the powder alkaline, it relieves the sensitiveness so that the patient will more thoroughly masticate his food and bring about a normal condition of the saliva, and he will not require the alkaline powder any length of time.

